# Innovative Supported Membranes for Ion Mobility Spectrometer (IMS) Sample Introduction Systems with High Permeability Relative to Toxic Agents in Air (TAAs)

**DOI:** 10.3390/ma18020281

**Published:** 2025-01-10

**Authors:** Monika Wiśnik-Sawka, Wojciech Fabianowski, Dorota Gajda

**Affiliations:** Military Institute of Chemistry and Radiometry, gen A. Chruściela “Montera” 105, 00-910 Warsaw, Poland; sekretariat@wichir.waw.pl

**Keywords:** polydimethylsiloxane-supported membranes, ion mobility spectrometry, higher permeability of toxic agents in air

## Abstract

One of the main objectives of the ion mobility spectrometry (IMS) technique is to reduce moisture in detection systems, which causes the formation of ion clusters and ion water and a reduction in formed clusters’ activity. Thus, one of the methods limiting moisture in a sampling injection system is to use hydrophobic polymeric membranes. The use of membranes with high permeability relative to the analysed organic compounds is required, including toxic agents in air (TAAs). Such requirements align with those of polydimethylsiloxane (PDMS) membranes. Unfortunately, thin PDMS membranes are not mechanically resistant. In this study, relatively thin PDMS membranes were reinforced with fine mesh fabric supports. These supports were chemically modified with selected oligoglycol derivatives and finally coated with PDMS. The obtained membranes were tested for water permeability and TAA simulants.

## 1. Introduction

Polymeric materials have a wide range of applications in various fields due to their diverse properties. One of the applications of polymers is membrane production, which is used to separate substances between two phases due to their properties [1]. Membrane modification causes an increase or a decrease in permeability with respect to selected substances via polymer structures [2,3].

Toxic agents in air (TAAs) are a group of highly toxic compounds, and their physiological classification is as follows: nerve agents (soman, sarin, tabun, and Vx group), blistering agents (sulphur mustard, nitrogen mustard, and lewisite), blood agents (hydrogen cyanide and cyanogen chloride), and choking agents (phosgene and diphosgene) [4]. Protection from these substances on the battlefield is provided by protective clothing, which comprises a mechanical barrier against the contact of TAAs with the soldier’s body. Research on materials with reduced permeability to TAAs is the subject of a large number of scientific works and patents [5,6,7]. The other method of protection is the fast detection and identification of these compounds at the point of their dispersion. This is particularly important because it constrains contact with toxic substances. Current research is focused on achieving even lower detection thresholds for TAAs.

The ion mobility spectrometry (IMS) technique is characterised by the fast generation of an analytical signal (time constant of several seconds) and continuous operation. The number of organic compounds that could possibly be detected and analysed utilising this technique is large. Hence, IMS has wide applications in forensics and military purposes [8,9,10,11].

IMS detectors are widely utilised for the rapid detection of high-energy materials (HEMs), TAAs, or drugs [8,12,13]. IMS detectors have found applications in both laboratory equipment and portable chemical compound detectors. They are characterised by high sensitivity, with a limit of detection measured in ppb, and short measurement times—less than one minute per whole cycle. For this reason, IMS detectors are also used in analytical laboratories, e.g., for food sample analyses [10,14,15]. Instruments that use the IMS detector can be equipped with a sample introduction system (SIS). Often, a polymer membrane is used as a component of this system. It restricts the access of the analysed gas and components and, in particular, eliminates the access of water vapour to the ionisation tube [16,17,18].

One of the challenges in designing IMS detection systems is to reduce moisture levels that are introduced to the sample injection system. The high level of moisture causes the formation of hydrated ion clusters, which decreases analysed ion clusters and their reactivity. High humidity levels significantly affect the ionisation capacity of the analyte. Water molecules may react with the analyte and form hydrated ion clusters that hinder the detection of organic matter. Moreover, the higher the analyte’s humidity, the lower the intensity of the peak signal [19,20,21].

Applying hydrophobic polymeric membranes in the sample injection system is one of the methods for achieving a reduction in water content in the analyte. Two types of membranes are utilised in the IMS technique: track-etched (TE) membranes and membranes made of polydimethylsiloxane (PDMS). Unfortunately, thin PDMS membranes are not mechanically resistant. To increase mechanical resistance, membranes are reinforced with fine mesh fabric support [22,23].

One approach to lowering the detection threshold for IMS detectors is the usage of polymer membranes in SIS, which not only limit moisture content but are also characterised by increased permeability relative to selected organic compounds. PDMS membranes are widely used in IMS techniques due to their high hydrophobicity and simple membrane modification [12,13,14,15,24,25,26,27,28,29,30].

The process of chemical compound permeation in solid PDMSs is complex and determined by penetrant–polymer interactions [22]. The mechanism of diffusion is based on the accommodation (absorption) of the penetrant and the creation of voids to ensure that the penetrant can pass through, i.e., be transported in the polymer [23]. 

The interaction of water with PDMS has been extensively discussed, particularly in terms of the statistical–mechanical binding theory of water molecules and the theory of water polycondensation [22,31,32,33,34,35,36,37,38,39,40]. In the case of water, single-molecule diffusion does not always occur. Water undergoes polycondensation, resulting in the diffusion of multimolecular conglomerates in the material. 

Studies [22,32] have shown that the diffusion coefficient is closely related to water activity, which is associated with a reduction in water permeation through a PDMS membrane. Banerjee et al. [38,39] have investigated water permeation [38,39,40] and have also investigated permeation for dimethyl methylphosphonate (DMMP) used as a TAA simulant. It was found that at room temperature, water permeates slower than DMMP.

In this study, membrane supports were modified with polydimethylsiloxane (PDMS) and additives. During the research, supports such as irradiated track-etched poly(ethylene terephthalate) (TE-PET) film, Cordura^®^ (Invista, Wichita, KS, USA), nylon, and elastane were investigated. Additionally, the membrane’s properties were modified with additives such as the oligomers of poly(ethylene glycol) (PEG), polypropylene glycol oligomer (PPG), and calix[6]arene to increase TAA permeability. The membranes obtained were subjected to liquid water permeability tests, and their permeability relative to water vapour was also determined. A drift tube ion mobility spectrometer (DT IMS) was applied to measure the number and amount of membrane impurities. The main objective of the studies was to obtain mechanically robust PDMS membranes with limited permeability relative to water and high permeability relative to TAAs. These membranes would be cheaper, attractive alternatives for track-etched membranes, which are widely used in the SIS of IMS detectors.

## 2. Materials and Methods

### 2.1. Chemicals

For membrane preparation, polydimethylsilicone PDMS (Sylgard 184, Dow Corning, Midland, MI, United States) and two elastomer components were used [41]. A mixture of hexane (purity 95%, Sigma-Aldrich, St. Louis, MO, US) and isopropanol (ACS reagent, ≥99.5%, Sigma-Aldrich) was used as a solvent for PDMS. Different admixtures were added to this mixture. The admixtures are shown in Table 1.

PDMS polymers are highly hydrophobic, electro-insulating materials, and they are transparent within the UV–Vis range. After cross-linking, they are resistant relative to organic solvents and high temperatures. These polymers are completely inert with regard to human health, which is why they are used in the pharmaceutical or food industry. Membranes made of PDMSs are commonly used for the separation of many organic compounds.

Membranes are characterised by specific parameters, which include the permeability P, solubility S, and the diffusion coefficient D of the compound in the material. The permeability depends on D and S and is described using the following Equation (1):

P = D · S (1)

In order to increase the diffusion coefficient, relatively thin PDMS membranes are required. These membranes must maintain mechanical strength and resistance to varying pressures [42,43]. Several different methods can be applied for the preparation of thin PDMS membranes, including Langmuir–Blodgett transfer (LBT) [44,45,46], casting a drop of a polymer solution onto a water surface [47,48,49,50], or casting drops from dilute solutions.

The solubility of an analysed compound in a polymer material can be increased by adding admixtures. These can be organic substances or inorganic molecules dissolved in the polymer or those incorporated into the polymer structure. The admixtures must demonstrate chemical similarity to the tested compounds.

In the first step of the research, the properties of thin PDMS membranes cast from dilute solutions onto thin, porous grids were tested as reinforcing supports. These supports should allow the obtainment of thin PDMS membranes with thicknesses of a few micrometres. Moreover, the obtained membrane must be relatively mechanically robust.

All supports were carefully washed with a mixture of hexane and isopropanol prior to PDMS solution casting. Subsequently, they were dried at 40℃ and placed on a levelled glass plate.

The membrane’s properties were characterised by chemicals such as the following: betanin (red beetroot extract diluted with dextrin) (Aldrich) and TAA simulants: dipropylene glycol monomethyl ether (DPMG) (mixture of isomers for synthesis, Millipore); dimethyl methylphosphonate (DMMP) (purity ≥ 97%, Sigma-Aldrich); and methyl salicylate (MS) (purity ≥ 99%, Sigma-Aldrich).

### 2.2. Membrane Preparation

#### 2.2.1. Membrane Supports

Four supports were used for membrane preparation: TE-PET film, Cordura^®^, elastane fabrics, and nylon.

**Figure 1:**
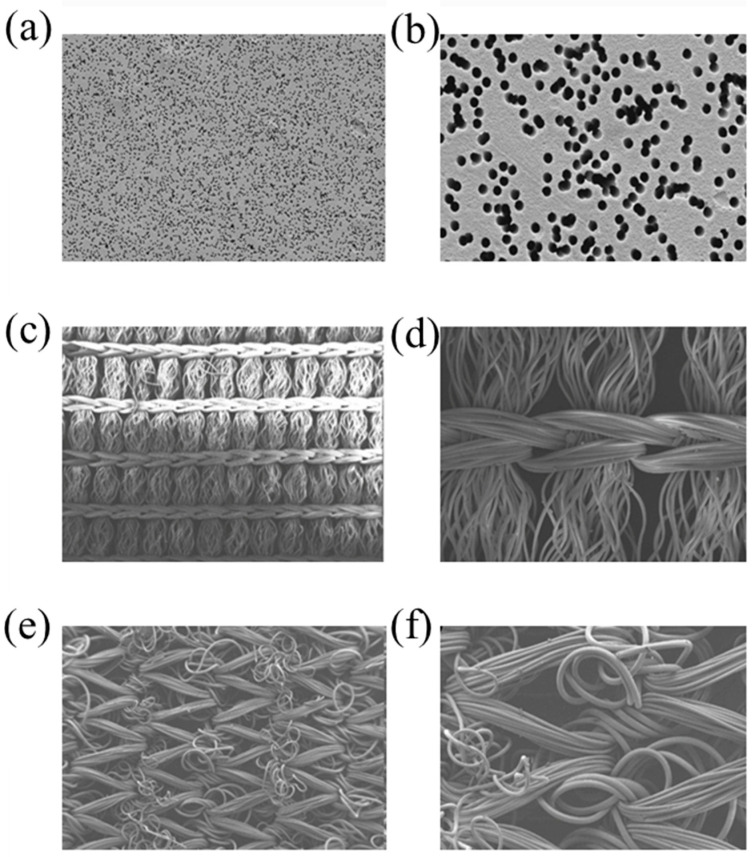
SEM picture of TE-PET supports: (**a**) 0.2 µm, (**b**) 1.0 µm; Cordura^®^: (**c**) magnification 50×, (**d**) magnification 200×; and elastane: (**e**) magnification 100×, (**f**) magnification; 1000×.

Poly(ethylene terephthalate) (PET) films were irradiated with accelerated Kr ions (energy E of 250 MeV) and subsequently etched with acids (these films were delivered by the Institute of Chemistry and Nuclear Technology (INCT), Warsaw, Poland). The utilised technique made it possible to produce primers with different pore diameters: 0.2, 0.4, 1.0, and 1.3 µm (Figure 1a,b).

Cordura^®^ (Dow Corning) [51] (Figure 1c,d) is an abrasion-resistant fabric made of polyamides, with a polyurethane coating primed with Teflon.

The elastane fabrics comprise polyether–polyurethane copolymers (The Lycra Company, Wilmington, DE, USA) [52] (Figure 1e,f).

Nylon [53] comprised 63% polyamide and 37% elastane (Ferax Sp. z o.o.), with fabric weave thicknesses of 15 and 8 den.

TE-PET film and Cordura^®^ were cut into 6 cm × 6 cm squares. Elastane fabrics and nylon were cut into 5.5 cm × 5.5 cm squares. All supports were placed on a levelled glass plate, and a PDMS/hexane solution was applied using a syringe. This method allowed for the covering of these supports with a controlled volume of applied solution and thorough distributions over the surface. After 24 h of drying at room temperature, the membranes were placed in an oven (120 °C) for 2 h.

#### 2.2.2. Membrane Characterisation

Table 2 shows data on the prepared PDMS/support membranes. The membranes’ thicknesses were calculated using Equation (2) [41]:

(2) $$d_{m}=\frac{\Delta m}{\rho_{PDMS}A}$$

where *d_m_* denotes the thickness of membranes [cm]; Δ*m* denotes a change in support mass [g]; *ρ_PDMS_* denotes the PDMS density [1.03 g/cm^3^]; and *A* denotes the support area [cm^2^].

Nylon with weave thicknesses of 8 and 15 denier was used to prepare doped membranes. The PDMS layer was modified via PEG or PPG with different molecular weights or via Calixarene. PDMS membranes with additives were prepared by mixing an isopropanol solution of PEG, PPG, and Calixarene with a hexane polysilicon solution in a weight ratio of 1:5 and poured onto the selected microporous support mesh. The diversity in membrane thickness was obtained by changing the volume (1–5 cm^3^) of the casting solution: 25% *w*/*w* PDMS/hexane solution or 2.8% *w*/*w* PDMS/hexane solution. Membrane data are shown in Table 3. Membrane thicknesses were determined in the same manner as for undoped membranes.

### 2.3. Experimental Methods

#### 2.3.1. Membrane Leakage Test

**Figure 2:**
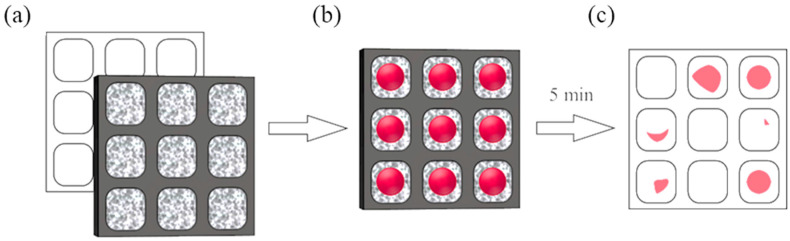
Liquid water permeability testing of the supported PDMS membranes with a red dye betanin water solution. (**a**) Membranes placed on a sheet of paper; (**b**) membranes with drops of betanin red dye; (**c**) sheet of paper after membrane removal for 5 min.

The obtained PDMS membranes were tested for permeability relative to liquid water in a simple laboratory procedure (Figure 2). The flat membranes were placed in a rack’s windows. In the next step, the rack was placed on a white sheet of paper. A betanin solution was applied to each of the tested membranes. The areas of the applied solution were determined. After 5 min, the rack was removed from the sheet of paper and traces from the red dye were observed. The tightness of the membrane was defined as the ratio of the area of the stain on the paper (A_S_) to the area of the dye applied to the membrane (A_D_).

#### 2.3.2. Gas Permeability

The tight membranes were tested relative to water vapour levels and TAA permeability. In this research, a variation of the ASTM E96 method, e.g., the upright cup method, was used [54]. The investigated membrane was placed in the cap to cover the hollow space. In the first stage, the vials were filled with 5 mL of water. The vial was twisted and weighed. Subsequently, the vials were left in an incubator at 25 °C. The samples were weighed at different times. This experiment allowed for the determination of the mass loss curve.

These modified membranes were characterised using a static method. Such a solution is commonly used to prepare permeation standards used to obtain calibration curves for the IMS detector under study [55,56,57].

#### 2.3.3. Membrane’s Self-Cleaning

**Figure 3:**
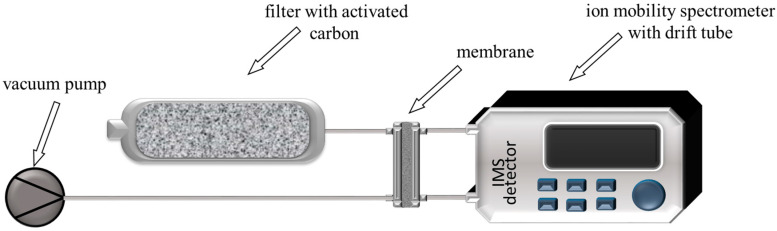
System for measuring membrane self-cleaning.

Moreover, the membrane’s ability to clean itself with airflow was investigated. Ion mobility spectrometry was utilised to observe impurity removal from the tested membranes. Figure 3 shows the membrane self-cleaning system.

An ion mobility spectrometer with a drift tube was used as a rapid analysis element (the detector was designed and commissioned at the Military Institute of Chemistry and Radiometry). To ensure that the membrane was washed with clean air, an activated carbon filter was connected to the inlet of the diffusion cell. The composition of the permeate was analysed continuously in the IMS detector.

#### 2.3.4. Membrane Permeability Relative to TAA

The membrane’s permeability relative to TAA was observed using the colorimetric method, which is utilised for TAA identification in the Armed Forces. The simulants are a group of compounds with similar physicochemical properties to TAAs; fortunately, they do not exhibit such high toxicity.

For the simulants, three chemicals were chosen:

- DPMG and MS as H-type blister agents (simulation of sulphur mustard);
- DMMP as a G-type nerve agent (simulation of phosphoroorganic TAAs such as tabun, sarin, or soman).

The investigated membrane was placed into the cap to cover a hollow. One drop of the DPMG, MS, or DMMP (0.5 cm^3^) was placed inside the vial and capped with a membrane stopper. TAA was detected using a paper chemical agent detector (indicator paper—3-way liquid, adhesive-backed No. 6665-21-858-8494). The paper was cut and placed onto a Petri dish. The vial and Petri dish were placed in a desiccator. The degree of permeation was tested by taking a photo of the paper. The analysis was carried out based on photographs taken at specific time intervals. The photographs were subjected to colour analyses in a graphics programme. The colour change was determined by determining the values of the colour components red (R), green (G), blue (B), hue (H), saturation (S), and value (V), which were determined from several points in the photo. Based on the values, a colour was generated, which is illustrated in the last column of the Table 4. Due to the many components, the colour change analysis was carried out based on the observation of the generated colour.

## 3. Results

**Figure 4:**
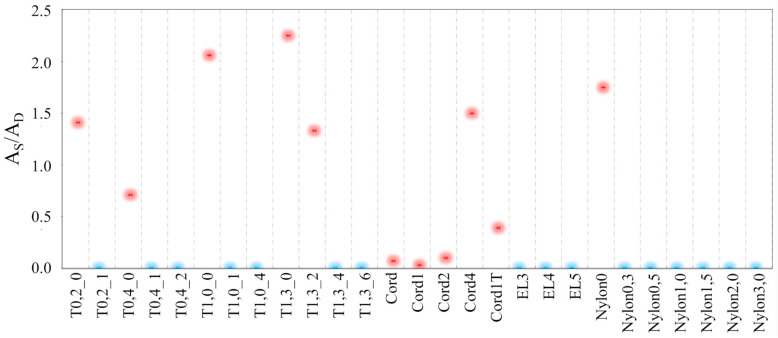
Liquid water permeability testing of PDMS membranes cast on four supports: A_S_—the area of the red spot on the paper; A_D_—the area of the dye applied to the membrane. Blue color indicates sealed membranes.

In Figure 4, data for the liquid water permeability testing of PDMS membranes cast on different supports are presented. If there were any dye spots on a sheet of paper after membrane removal, the A_S_/A_D_ ratio was determined; if A_S_ = 0, the membrane did not experience leakage. An A_s_ value higher than 0 excludes a membrane’s further use in this research.

**Figure 5:**
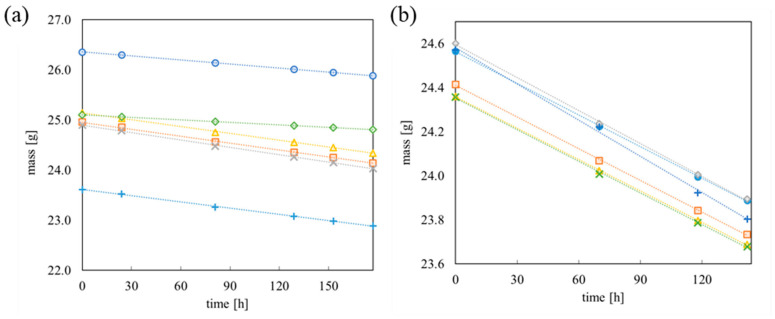
Weight loss of water in the glass vials capped with (**a**) PDMS/elastane membranes with different PDMS thicknesses: x—124 µm; ■—140 µm; +—170 µm; ▲—200 µm; ●—353 µm; ♦—426 µm. (**b**) PDMS/nylon 15 den membranes with different PDMS thicknesses: ●—0.0 µm; ■—2.7 µm; ♦—4.7 µm; ▲—6.1 µm; +—7.7 µm; x—9.9 µm.

The most promising results were obtained for TE-PET membranes with pore sizes of 1.0 µm and 1.3 µm, which were modified with 2 or 4 mL of PDMS/hexane 10% *w*/*w* solution and all elastane- and nylon-reinforced membranes. Because TE-PET membranes were treated only as reference membranes, they were not tested in the subsequent research study. The Cordura^®^-supported membrane was highly permeable relative to water in tests with an aqueous solution of betanin (red dye). It is likely that the primers were poorly wetted with the polymer solution. This phenomenon may have caused the membrane’s leakage. All elastane and nylon membranes were tight; thus, they were tested for water vapour permeability. The results of the permeability to water vapour test are shown in Figure 5. During the measurement, the loss of the vial’s weight over time was recorded.

The same tests were carried out for membranes supported with elastane and 15-denier nylon.

**Figure 6:**
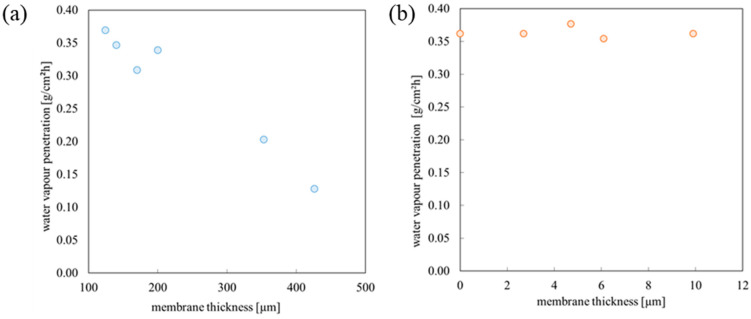
Water vapour penetration for membranes with different thicknesses: (**a**) PDMS/elastane membranes; (**b**) PDMS/nylon 15 den membranes.

The time dependence of water mass loss allows the determination of the permeability expressed in terms of the amount of water vapour passing through a unit area of membrane per unit time. Figure 6 shows the calculated water vapour penetration values for different membrane thicknesses.

The thickness of PDMS/elastane membranes was within the range of 100 μm to 400 μm. The research showed that for these membranes, water vapour permeability decreases from 0.12 to 0.37 gcm^−2^h^−1^. An increase in membrane thickness causes a decrease in permeability relative to water.

The thicknesses of PDMS/nylon membranes were within the range of 3.4 to 8.6 μm. For these membranes, the change in thickness was not substantial; therefore, permeability for each membrane was at similar levels—0.34 to 0.415 gcm^−2^h^−1^.

**Figure 7:**
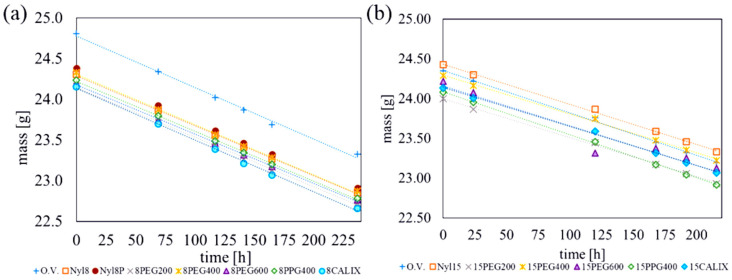
Weight loss of water in the vials capped with doped membranes: (**a**) PDMS/nylon 8 den; (**b**) PDMS/nylon 15 den.

The permeability value for water vapour was also determined for doped membranes. Changes in weight values over time for membranes on 8 den nylon and 15 den supports are shown in Figure 7.

The membranes obtained were characterised via a constant water vapour permeation value, as shown in Figure 5 and Figure 7. The water vapour concentration over the membrane was constant due to the stability of water emissions from the vial.

**Figure 8:**
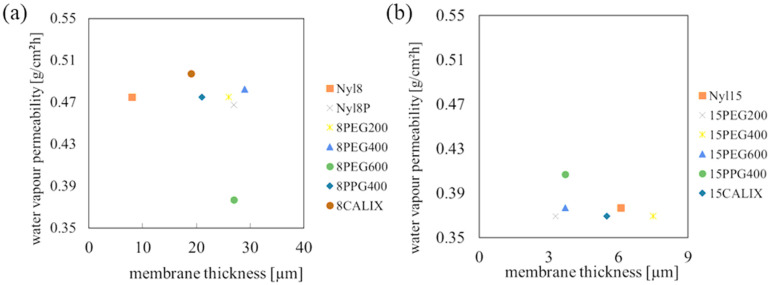
Water vapour permeability for membranes with different thicknesses: (**a**) PDMS/nylon 8 den; (**b**) PDMS/nylon 15 den.

For the membranes supported via 8 den nylon, the permeability to water vapour was reduced only for the 8PEG600 membrane (Figure 8a). For the membranes modified with Calixarene, the permeability to water was even higher than for the undoped reinforced membrane itself. For the membrane supported with 15-denier nylon (Figure 8b), the addition of PPG400 increases the permeability to water. No significant change in permeation was observed for other admixtures. It is worth noting that the addition of PEG, PPG, or Calixarene has an influence on the water vapour permeability (WVP) value (Figure 8).

**Figure 9:**
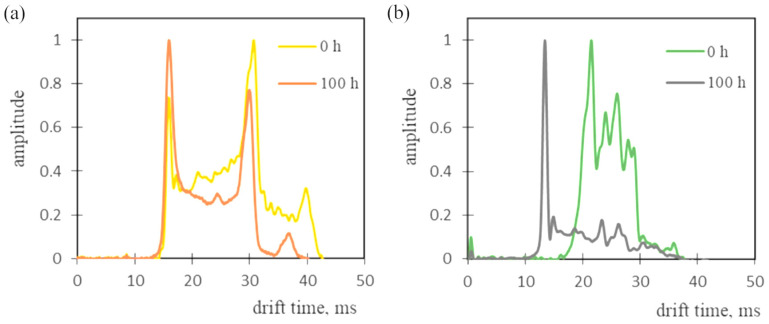
Drift time spectra obtained during membrane cleaning: (**a**) Nyl15; (**b**) 8PEG600.

Two membranes—8PEG600 and Nyl15—were tested with respect to their airflow self-cleaning ability. For this purpose, a system with an ion mobility spectrometer was applied (Figure 3). Each membrane tested was mounted in the diffusion cell of the system, and the drift time spectrum was recorded as soon as the measurement began. The results of the experiment are shown in Figure 9.

Both membranes were heavily contaminated and were therefore cleaned in an air stream for approximately 100 h. The membrane modified with 8PEG600 was almost impurity-free; for that reason, it was applied in further tests.

The 8PEG600 membrane was selected for the permeability studies of DPMG, MS, and DMMP. This membrane was chosen because of its low water vapour permeability and strength. The results of the study are shown in Table 4.

Research on permeability relative to TAAs was carried out analogically compared to permeability relative to water. Indicator papers are the simplest means used for the detection of the presence of poisonous TAAs during military operations (including on the battlefield during wartime). The biggest advantage of using indicator papers is that they are relatively easy to use. The change in colour of the paper provides the soldier with clear and legible information about the dispersion of poisonous substances in the surrounding atmosphere. The colour change analysis is described in Section 2.3.4. The values of the colour components are shown in Table 4. Regrettably, indicator papers have relatively low sensitivity; nevertheless, they are accepted by the Armed Forces as an indicator for the preliminary detection of TAAs.

Permeability tests for DPMG, MS, and DMMP were carried out in a similar manner as for water. A drop of DPMG, MS, or DMMP (0.5 cm^3^) was deposited at the vial’s bottom, and the investigated membrane was placed in the vial cup. The indicator’s paper was located on the cup’s surface. Taking into account the very low vapour pressure of the tested MS (14.8 Pa [58]), DMMP (<0.1 Pa [59]), and DPMG (53.3 Pa [60]) compared to a water vapour pressure of VP = 3169 Pa [61], the change in the vial’s mass could be registered after several dozen hours. For this reason, research on DPMG, MS, and DMMP permeability was carried out using indicator papers.

TAA colorimetric indices showed colour changes after exposure to all tested DPMG, MS, and DMMP pairs. For MS, the most intense colour occurred up until 240 h; nevertheless, the indicator paper began to redden just after 24 h. DMMP and MS caused a change in colour within 24–72 h. The colouration obtained for DPMG occurred rapidly. Any intensity change in colour was not registered after 24 h.

Taking into account the very low vapour pressure of the tested simulants of TAAs and the appearance of the colour reaction on the indicator, these observations prove that the selected membrane permeability increased after modification, and it was high for all tested DPMG, MS, and DMMP. 

## 4. Conclusions

This research study focused on finding more cost-effective and attractive membranes, compared to track-etched membranes that are produced only by the Joint Institute for Nuclear Research in Dubna (irradiated in Dubna and etched with acids at the Institute of Nuclear Chemistry and Technology, Warsaw, Poland). Given the scarcity of the alternative membranes, the purchase of track-etched membranes is currently almost impossible.

Three supports were selected (Cordura^®^, nylon, and elastane) for further modification with PDMS in order to obtain waterproof and mechanically robust membranes.

Studies have shown that there is no justification for modifying Cordura^®^ material. This material is airtight and hydrophobic, but it loses its properties and becomes permeable relative to water after applied modifications. In the case of unmodified membranes, the most promising results were observed for membranes supported with elastane and nylon. After PDMS modification, these supports were waterproof.

Apart from pure PDMS, chosen supports were also modified with a mixture of PDMS with additives (PEG, PPG, and calix[6]arene) to increase TAA permeability.

The thickness of PDMS/nylon was within the range of 3.4 to 8.6 µm, and in this range, changes in water vapour permeability have not been observed. PDMS/elastane membranes were thicker, and significant changes in water permeability within the membrane thickness range of 100 µm to 400 µm were observed.

Of all modified membranes, the most effective was 8PEG600, for which the lowest degree of water vapour permeability was observed. This membrane also exhibited the highest self-cleaning ability.

Preliminary tests performed for DPMG, MS, and DMMP using the indicator papers showed that the selected membrane exhibits increased permeability relative to TAAs. This is due to the fact that the sensitivity of papers for simulants is lower than for TAAs.

In a PhD thesis, the emissions of DMMP through pure PDMS membranes, which appeared after 100 h (35 °C) in static conditions, were described [62]. This is why we can conclude that the obtained membrane (PDMS with PEG, reinforced with 8 den nylon) is characterised by its higher permeability relative to DMMP: the emissions of DMMP through the modified membrane appear after 24 h (25 °C) in static conditions.

This study proved that nylon used as a support could be a low-cost, readily available material that can provide adequate backing for the membrane with respect to specific membrane properties. This study presents results that could become a significant foundation for research on materials with increased permeability relative to highly toxic substances such as TAAs.

## Figures and Tables

**Table 1:** Admixtures for membrane modification.

Admixture	Molecular Mass [g/mol]
PEG (Sigma-Aldrich)	200 (BioUltra, 200)
	400 (BioUltra, 400)
	600 (BioUltra, 600)
PPG (P 400, Sigma-Aldrich)	400
Calix[6]arene (purity 97%, Sigma-Aldrich).	636.7

**Table 2:** Data for the preparation of different PDMS/support membranes.

Support	PDMS Concentration [% *w*/*w*]	Pore Diameter [µm]	PDMS Volume [mL]	PDMS Membrane Thickness [µm]	Abbreviation
TE-PET film	10	0.2	0	0	T0,2_0
			1	17	T0,2_1
		0.4	0	0	T0,4_0
			1	16	T0,4_1
			2	15	T0,4_2
		1	0	0	T1,0_0
			1	15	T1,0_1
			4	24	T1,0_4
		1.3	1	0	T1,3_0
			2	14	T1,3_2
			4	27	T1,3_4
			6	34	T1,3_6
Cordura^®^	44	(-)	0	0	Cord
			1	57	Cord1
			2	110	Cord2
			4	41	Cord4
			1	-	Cord1T
Elastane	25.5	(-)	3	150	EL3
			4	200	EL4
			5	360	EL5
Nylon	2.9	15 den	0.0	0	Nylon0
			0.3	3.2	Nylon0,3
			0.5	4.6	Nylon0,5
			1.0	5.7	Nylon1,0
			1.5	6.0	Nylon1,5
			2.0	7.2	Nylon2,0
			3.0	8.6	Nylon3,0

**Table 3:** Data for doped PDMS membranes supported with a fine nylon mesh.

Membrane Composition	Thickness [µm]	Molecular Weight of Additives [g/mol]	Abbreviation
Open vial	0	(-)	O.V.
Nylon 8 den	18	(-)	Nyl8
Nylon8/PDMS	27	(-)	Nyl8P
Nylon8/PDMS + PEG	26	200	8PEG200
Nylon8/PDMS + PEG	29	400	8PEG400
Nylon8/PDMS + PEG	27	600	8PEG600
Nylon8/PDMS + PPG	27	400	8PPG400
Nylon8/PDMS + Calix	19	264	8CALIX
Nylon15/PDMS	6.1	(-)	Nyl15
Nylon15/PDMS + PEG	3.3	200	15PEG200
Nylon15/PDMS + PEG	7.5	400	15PEG400
Nylon15/PDMS + PEG	3.7	600	15PEG600
Nylon15/PDMS + PPG	3.7	400	15PPG400
Nylon15/PDMS + Calix	5.0	264	15CALIX

**Table 4:** Results of the changes in colorimetric chemical agent detectors after a long period of contact with DPMG, MS, or DMMP in the gas phase. Red (R), green (G), blue (B), hue (H), saturation (S), value (V), and white light components.

Substance	Exposure Time [h]	R	G	B	H	S	V	View
MS	0	54	45	31	50	24	75	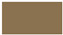
	24	52	48	51	312	7.5	52	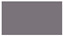
	72	53	45	43	14	18	53	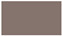
	240	56	43	40	11	28	56	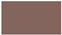
DMMP	0	55	46	31	50	25	77	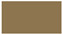
	24	50	45	31	43	36	50	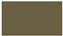
	72	55	51	30	43	49	59	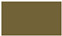
	240	56	51	33	45	40	56	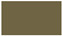
DPMG	0	57	49	34	53	23	77	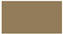
	24	54	55	53	72	3	55	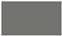
	72	56	55	47	54	16	56	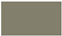
	240	57	58	57	100	2	58	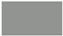

## Data Availability

Data are unavailable due to privacy.

## DURC Statement

Current research is limited to the material science and chemical engineering, which is beneficial for future design of equipment for first responders and does not pose a threat to public health or national security. Authors acknowledge the dual use potential of the research involving preparation of innovative supported membranes and confirm that all necessary precautions have been taken to prevent potential misuse. As an ethical responsibility, authors strictly adhere to relevant national and international laws about DURC. Authors advocate for responsible deployment, ethical considerations, regulatory compliance, and transparent reporting to mitigate misuse risks and foster beneficial outcomes.
